# Validation and dyadic invariance of the Infertility Concerns Questionnaire among Brazilian couples

**DOI:** 10.3389/fpsyg.2025.1504554

**Published:** 2025-06-27

**Authors:** Victor Zaia, Paola Gremigni, Giulia Casu

**Affiliations:** ^1^Postgraduate Program in Health Sciences, Centro Universitário FMABC, Santo André, Brazil; ^2^Ideia Fértil Institute of Reproductive Health, Centro Universitário FMABC, Santo André, Brazil; ^3^Department of Psychology, University of Bologna, Bologna, Italy

**Keywords:** infertility, desire for parenthood, communication, psychometric validation, dyadic invariance, assisted reproductive technology

## Abstract

**Introduction:**

Infertility often brings profound emotional challenges, intertwining the desire for parenthood with the complexities of communication within and beyond the couple. Existing scales fall short of fully capturing these nuances, prompting the development of the Infertility Concern Questionnaire (ICQ), which uniquely addresses both the parental desire and communication dimensions.

**Methods:**

The ICQ was completed by 350 opposite-sex infertile couples from Brazil, aged 22–58 years, who were undergoing fertility treatment. Both partners of all couples completed the ICQ, and a subsample of 168 couples also completed a measure of infertility-related stress. The factor structure of the ICQ was evaluated using confirmatory factor analysis, and measurement invariance across couple members was tested within a dyadic framework.

**Results:**

Configural, metric, partial scalar, and strict measurement invariance was supported for the ICQ’s theoretical two-factor model of infertility concerns as made of parental (PAR - Examines the central role that the theme of procreation assumes in the construction of individual identity) and communication (COM - Investigates the significance of interpersonal sharing of reflections and experiences related to the decision to have—or not to have—children) dimensions. No between-partner differences were found in ICQ latent factor means. Reliability was found to be acceptable for both dimensions across genders. PAR correlated positively with intrapersonal infertility-related stress in both males and females, while COM correlated negatively with interpersonal infertility-related stress in females. Significant associations between ICQ scores and type of infertility, history of miscarriage, and treatment failures were observed among females.

**Discussion:**

The findings provide initial evidence of the ICQ’s validity and reliability, suggesting its potential use by professionals in fertility treatment settings to better assess and address the distinct emotional and communicative concerns of individuals and couples experiencing infertility.

## 1 Introduction

The World Health Organization (WHO) estimates that 15% of couples of reproductive age globally experience infertility (WHO WHO, 2006). Infertility negatively impacts individuals both intrapersonally and interpersonally (Casu and Gremigni, 2016). Intrapersonally, it affects quality of life, life satisfaction, mental health, self-esteem, and sexuality. Many individuals also grapple with guilt and a deep sense of failure, reflecting the heavy emotional toll infertility imposes on one personal sense of self (Ho et al., 2020; Taebi et al., 2021; Luca et al., 2021). Interpersonally, infertility often leads to social stigma and pressure to achieve parenthood, which can deteriorate social relationships, reduce access to social support, and lead to social isolation (Daniluk, 1988; Shreffler et al., 2020; Chamorro et al., 2024; Kiesswetter et al., 2020). Therefore, addressing both the internal and social challenges of infertility is essential when assessing psychological adjustment in both men and women (Braverman et al., 2024; Sharma and Shrivastava, 2022; Gameiro et al., 2015).

The diagnosis of infertility typically occurs after a year of unsuccessful attempts to conceive and marks the beginning of a treatment journey (Boivin et al., 2023), which can reshape life decisions and place the desire for parenthood at the forefront of personal priorities, profoundly influencing and altering various aspects of infertile individuals’ lives (Massarotti et al., 2019; Zurlo et al., 2023). To address infertility, more than half of affected couples pursue medical interventions, such as assisted reproductive technology (ART) treatments (Boivin et al., 2007). ART encompasses a variety of medical and biological techniques to facilitate fertilization and support pregnancy, both *in vivo* and *in vitro* (Jain and Singh, 2024). However, ART does not guarantee success, with success rates ranging between 30 and 40%, depending on factors like infertility type, patient age, and the specific ART methods employed (Präg et al., 2017). The physical, financial, emotional, and time-consuming burdens of ART, alongside its uncertain outcomes, introduce significant additional stress (WHO WHO, 2006; Taebi et al., 2021; Ozturk et al., 2021) and contribute to a substantial percentage of couples discontinuing treatment prematurely (Pedro et al., 2017; Assaysh-Oberg et al., 2023; Shen et al., 2024). Women, in particular, tend to experience greater emotional distress throughout ART treatment (Bose et al., 2021; Tavousi et al., 2022).

The desire for parenthood is a complex, deeply personal aspiration that varies widely among individuals and cultures. Parenthood is frequently associated with personal fulfillment, the continuation of family traditions and values, and the desire to nurture and raise a child (Miscioscia et al., 2017). Unfulfilled parenthood aspirations can lead to feelings of sadness, frustration, and even depression (Assaysh-Oberg et al., 2023; Batz et al., 2023; Biggs et al., 2024; Canada and Schover, 2012). Conversely, the prospect of becoming a parent can bring joy and a sense of purpose. Cultural norms and societal expectations play a central role in shaping the desire for parenthood. In many cultures, having children is seen as a natural and essential part of life, while in others, greater emphasis may be placed on personal or professional achievements (Cetre et al., 2016; Järvinen, 1998; Harkness and Super, 2002). Furthermore, biological instincts and the natural drive to reproduce, along with life circumstances such as financial stability, relationship status, and career goals, also influence the desire for parenthood and decisions around it (Baiocco and Laghi, 2013; Vignoli et al., 2022; Beaujouan, 2020). For many individuals, the desire to become a parent is not only about personal fulfillment but also about the emotional and relational wish to share the parenting experience with a partner, other family members, or close friends (Miscioscia et al., 2017; Baiocco and Laghi, 2013; Miller, 1995).

However, many individuals experience difficulty discussing infertility with people outside their family, and some even find it hard to talk about it with their spouses (Zurlo et al., 2023; Sormunen et al., 2018). These communication barriers often increase emotional distress and contribute to social isolation, making it crucial to identify those at risk of developing emotional problems due to these difficulties (Xie et al., 2022). Effective communication in the context of infertility goes beyond simply exchanging information; it involves providing emotional support, fostering mutual understanding, and working collaboratively to navigate the infertile condition (Jansen et al., 2022). Since infertility is frequently accompanied by feelings of grief, frustration, and anxiety, open communication between partners allows for the sharing of these emotions, enabling mutual support and reducing feelings of isolation. Discussing fears, hopes, and expectations openly can strengthen the emotional bond between partners, enhancing their resilience in coping with the challenges of infertility (Kielek-Rataj et al., 2020; Schmidt, 2006). Additionally, effective communication can assist partners in managing expectations and facilitating joint decisions about treatment and future plans (Gameiro and Finnigan, 2017).

Infertility often carries social stigma, which can shape how individuals discuss their condition with family, friends, and the broader community. Open and honest communication not only helps to reduce this stigma but also fosters a support network that is vital for emotional well-being (Taebi et al., 2021; Bose et al., 2021; Kielek-Rataj et al., 2020; Schmidt, 2006; Wang et al., 2024; Tang et al., 2022; Schmidt et al., 2005). When individuals can openly express the struggles of infertility and its treatments, they are more likely to receive social support, which plays a vital role in improving well-being (Massarotti et al., 2019; Sormunen et al., 2018; Schmidt, 2006; O’Connell et al., 2021; Chu et al., 2021). Therefore, cultivating open communication within relationships and social networks is essential for alleviating the emotional burden of infertility.

The desire for parenthood and communication difficulties are central concerns for many infertile individuals, influencing both their emotional well-being and their ability to cope with infertility and its treatment. Understanding these concerns in patients seeking ART can enable healthcare professionals, including psychologists, doctors, and nurses, to offer more tailored support strategies, addressing both the psychological and social needs of patients for more effective care.

In this context, various measures related to infertility experiences have been proposed, focusing on different psychological dimensions. The primary themes identified include the desire for parenthood, marital relationships, infertility-related stress, social relationships, anxiety, depression, and overall mental health (Tavousi et al., 2022; Nik Hazlina et al., 2022; Starc et al., 2019). General scales, such as the World Health Organization Well-Being Index (WHO-5) (Omani-Samani et al., 2019), the World Health Organization Quality of Life Assessment (WHOQOL) (WHO, 1995), and the Beck Depression Inventory (BDI) (Beck et al., 1961), were used also in the context of infertility. These are broad and comprehensive scales designed to assess mental health and quality of life, applicable to various conditions as well as the general population. Scales specifically tailored to the context of infertility were reviewed by Kitchen et al. (2017). These include, among others, the Fertility Problem Inventory (FPI) (Newton et al., 1999), the Fertility Problem Stress (FPS) (Abbey et al., 1992), the Fertility Quality of Life (FERTIQOL) (Boivin et al., 2011), the Infertility Questionnaire (IFQ) (Bernstein et al., 1985), and Parenthood Motivation List (PML) (van Balen and Trimbos-Kemper, 1995). Additionally, we considered the Infertility Related Stress Scale (IRSS) (Casu and Gremigni, 2016; Casu et al., 2021) a scale measuring stress specifically related to infertility, validated in the context of assisted reproductive technology.

While these existing scales provide valuable insights into various aspects of infertility, they often focus on specific themes such as stress, quality of life, or motivation. However, none comprehensively assess the unique interplay between the desire for parenthood and the communicative aspects of dealing with infertility. In this context, we propose the Infertility Concern Questionnaire (ICQ), a concise self-report scale designed to fill this gap. The ICQ stands apart from existing measures, such as the FPI, by addressing two dimensions that have not been fully explored together in previous instruments: the parental dimension, which focuses on the desire to become a parent, and the communicative dimension, which emphasizes the role of communication, both within and outside the couple, in enhancing well-being. What distinguishes the ICQ from other tools is its focus on the individual’s personal experience of infertility, rather than the consequences of infertility.

The present study aimed to develop and psychometrically test the Infertility Concern Questionnaire (ICQ) within the Brazilian context. We assessed content and face validity, validity based on factor structure and dyadic measurement invariance, reliability, and validity based on relations to other variables. Dyadic measurement invariance refers to the consistency of a questionnaire’s measurement properties across different members of dyads, such as romantic couples or parent–child pairs. This ensures that the questionnaire measures the same constructs in the same way for both members, enabling valid comparisons. Establishing dyadic invariance is crucial, as it ensures that any observed differences or similarities in responses reflect actual differences in the constructs being measured, rather than variations in how the questionnaire functions for each dyad member (Claxton et al., 2015). In this study, we tested dyadic invariance across males and females in infertile couples to ensure that the ICQ measures infertility concerns consistently for both partners.

## 2 Materials and methods

### 2.1 Participants and procedures

This cross-sectional study was conducted with couples undergoing ART at the Ideia Fértil Institute, Center for Human Reproduction and Genetics, located at Centro Universitário FMABC/Faculdade de Medicina do ABC in Santo André, Brazil. Participants were required to be at least 18 years old and have started ART treatment. Exclusion criteria included patients diagnosed with cancer, those pursuing ART for fertility preservation, single-parent families, and same-sex couples, as their reasons for treatment were not related to infertility.

All couples were personally invited to participate in the study by the first author. They were informed about the research details, and those agreeing to participate provided written informed consent. It was emphasized that participation was voluntary, and withdrawal or refusal to participate would have no impact on their treatment. Data collection was conducted using paper-and-pencil forms, with participants’ anonymity maintained.

A total of 376 couples attending the infertility clinic who met the inclusion criteria were invited to participate in the study. Of these, 350 couples (93%) agreed to participate and completed the questionnaire, while the remaining couples declined due to lack of interest. Participants were between 22 and 58 years old (mean age: women = 34.78 ± 4.81; men = 37.28 ± 6.31), and 50% were women. Both members of all couples were administered the Infertility Concern Questionnaire (ICQ), and approximately half of the couples (*n* = 168), randomly selected, were also given the Infertility Related Stress Scale–Brazilian Portuguese (IRSS-BP). Sociodemographic and infertility-related characteristics were collected from all participants. Sociodemographic information included gender, age, ethnicity, education, employment status, household income, and relationship length. Infertility-related information included the type of infertility (i.e., primary infertility, defined as never having conceived despite at least 12 months of attempting conception, or secondary infertility, defined as having had at least one prior conception but subsequently being unable to conceive after at least 12 months of trying), history of miscarriage, infertility diagnosis (i.e., male factor, female factor, both male and female factor, or unexplained), duration of infertility, type of current ART treatment, and whether couples had experienced previous failed ART cycles.

The study was approved by the Ethics Committee of the Centro Universitario FMABC (approval number 88192218.3.0000.0082/2.675.077) and adhered to the principles of the Helsinki Declaration.

### 2.2 Measures

#### 2.2.1 Infertility concern questionnaire

By reviewing existing infertility-specific scales, we identified several relevant instruments. The Fertility Problem Inventory (FPI, 46 items) assesses infertility-related stress across five domains: social, sexual, and relationship concerns, need for parenthood, and rejection of a childfree lifestyle (Newton et al., 1999); a Brazilian Portuguese version is available (Ribeiro, 2007). The Fertility Problem Stress Scale (FPS, 9 items) measures perceived infertility-related stress (Abbey et al., 1992), but no Brazilian Portuguese version exists. The Fertility Quality of Life tool (FERTIQOL, 24 items) evaluates the impact of infertility across four dimensions: emotional, relational, mind/body, and social (Boivin et al., 2011); a Brazilian Portuguese version is available from the original authors. The Infertility Questionnaire (IFQ, 21 items) focuses on self-esteem, blame/guilt, and sexuality (Bernstein et al., 1985), while the Parenthood Motivation List (PML, 18 items) assesses six motivations for having children (van Balen and Trimbos-Kemper, 1995); neither is available in Brazilian or Portuguese versions.

Based on this review, we identified a gap in the available instruments: the absence of a brief and accessible instrument in Brazilian Portuguese specifically designed to assess parenthood-related concerns and communication about the infertility experience in the context of medically assisted reproduction. To address this gap, we developed the Infertility Concern Questionnaire (ICQ) as a concise and easily applicable self-report scale, drawing inspiration from existing tools that assess experiences related to infertility (Newton et al., 1999; Boivin et al., 2011; Casu et al., 2021). We proposed 18 items aimed at exploring emotional and relational aspects, as well as individual and couple experiences associated with infertility, with particular attention to the desire for parenthood and communication about the infertility experience. Each item is rated on a 7-point Likert scale, ranging from 1 = “Completely false for me” to 7 = “Completely true for me.”

The ICQ items were submitted to a focus group consisting of seven Brazilian psychologists, all PhD students in health psychology, to assess content validity. Their objective was to evaluate the conceptual construction of the items, ensuring that each item accurately captured and measured the intended aspects as fully described. A minimum concordance rate of 80% was required for an item and its domain to be retained. Each focus group member reviewed the items to determine whether they aligned with the proposed instrument and to identify the appropriate domain – Parenthood (PAR) or Communication (COM) (Meijering et al., 2013). This level of agreement was achieved for 7 of the 18 originally proposed items, 4 of which pertained to the PAR domain (items 1, 2, 3, and 4) and 3 to the COM domain (items 5, 6, and 7). For the retained items, the group confirmed their face validity.

Subsequently, a focus group was conducted with a sample from the target population, invited by the authors. The group consisted of nine participants (56% women), aged between 20 and 45 years, with varied ethnic and socioeconomic backgrounds and the session lasted 1 h. The objective was to assess whether the items were clear, relevant, and meaningfully captured the participants’ experiences related to infertility. The participants did not suggest any modifications to the items and expressed full agreement with the ICQ format initially proposed by the expert focus group. Based on this feedback, the final version of the ICQ was approved for the next phase of validation and administered to the research target audience.

#### 2.2.2 Infertility related stress scale

The Infertility Related Stress Scale–Brazilian Portuguese (IRSS-BP) (Casu et al., 2021) is a 12-item self-report measure designed to assess the level of stress that infertility imposes on different aspects of life. This instrument has been validated in Brazilian Portuguese within the context of assisted reproductive treatment and is freely accessible. It consists of two 6-item scales referring to the intrapersonal (e.g., mental well-being) and interpersonal (e.g., friends) domains of life. Each item is rated on a 7-point scale from 1 (not at all) to 7 (a great deal). Higher scores indicate a higher level of infertility-related stress. McDonald’s *ω* in this study for the intrapersonal domain was 0.95 for males and 0.94 for females, and for the interpersonal domain was 0.96 for males and 0.96 for females.

### 2.3 Data analysis

Descriptive statistics were used to characterize the study sample. Using confirmatory factor analysis (CFA), the ICQ theoretical two-factor model and an alternative one-factor model were first tested in males and females separately. Dyadic measurement invariance of the selected model was then examined. To account for non-independence of observations at the factor and item levels, the factor model for males and females was connected through correlations between the latent factors, and error terms of parallel items were correlated between couple members. Increasingly restrictive models were compared that incrementally constrained model parameters to be equal across couple members, representing configural (equal factor structure), metric (equal factor loadings), scalar (equal intercepts), and strict (equal error variances) invariance. To test between-partner differences in latent factor means in case of achieved scalar invariance, we set the latent means as equal across couple members and compared this model against the scalar invariance model. Model fit was evaluated based root mean square error of approximation (RMSEA) and standardized residual mean root (SRMR), with values of ≤0.08 indicating acceptable fit, and comparative fit index (CFI), with values ≥ 0.90 as indicative of acceptable fit (Hu and Bentler, 1999). We evaluated measurement invariance based on both statistical and practical significance. Statistically, measurement invariance was considered achieved if nested models showed a non-significant decrease in model fit, as indicated by a non-significant *χ*^2^ difference test (Δ*χ*^2^). From a practical perspective, measurement invariance was considered achieved if the worsening of model fit was sufficiently small, as indicated by a decrement in CFI (ΔCFI) of ≤ 0.010, supplemented by increases in RMSEA (ΔRMSEA) and SRMR (ΔSRMR) of ≤0.015 and ≤0.010, respectively (Chen, 2007). If full measurement invariance was not achieved, partial measurement invariance was considered, which is viable when at least two items per latent construct have invariant parameters (Steenkamp and Baumgartner, 1998). To identify which items were responsible for the lack of invariance, equality constraints on the measurement parameters were sequentially relaxed. These nested models were compared using Δ*χ^2^* tests, with an adjusted α-level (Bonferroni correction). The effects coding identification method was used for measurement invariance testing, allowing latent parameters to be estimated in a non-arbitrary way by constraining the factor loadings to average 1 and the intercepts to sum to 0 (Little et al., 2006). The sample size was established *a priori* to ensure at least 5–10 observations for each estimated parameter in the baseline configural invariance model (Kline, 2016).

The estimation method used in the CFA was maximum likelihood (ML), which assumes multivariate normality. Since ML is more sensitive to deviations caused by kurtosis (Ryu, 2011), we evaluated multivariate normality using Mardia’s coefficient of multivariate kurtosis. The standardized value obtained was 8.022, which falls below the commonly accepted threshold of 10 for the application of ML estimation (Kline, 2016).

Reliability was assessed using McDonald’s *ω* (Gadermann et al., 2012), with acceptable values set at 0.70 or higher. Corrected item-total correlations were also calculated, with acceptable values greater than 0.30 (Nunnally and Bernstein, 1994).

To assess validity based on relations to other variables, Pearson’s correlations were computed to examine the relationships between the ICQ and IRSS-BP, while ANOVAs were performed to test associations between ICQ scores AND infertility-related characteristics separately in males and females. Effect size values were interpreted as follows: correlations of 0.20 were considered small, 0.30 medium, and 0.50 large; Cohen’s *d* values of 0.20, 0.50, and 0.80 indicated small, medium, and large effects, respectively (Cohen, 1988).

Significance level was set at *p* < 0.05. CFAs were conducted in Mplus 8.4. All other analyses were performed using IBM SPSS 29.

## 3 Results

### 3.1 Participants’ characteristics

The sample consisted of 350 opposite-sex infertile couples (i.e., 50% women), with participants aged between 22 and 58 years (mean age: women = 34.78 ± 4.81; men = 37.28 ± 6.31). About 60% of both males and females identified as being of European descent, while 27% reported mixed ethnicity. Half of the males and two thirds of the females were highly educated, holding a degree or post-degree, and the majority of both males (93.7%) and females (82.9%) were employed. More than half of the couples had a medium household income (53.1%) and had been together for 5–9 years (55.1%). Seventy percent of the couples experienced primary infertility, and 14% had experienced previous miscarriages. One-third of the couples had unexplained infertility, and two-thirds had been trying to conceive for more than 2 years. Around half of the couples had experienced previous failed ART cycles. Participants’ characteristics are detailed in Table 1.

**Table 1:** Sociodemographic and infertility-related characteristics of the participants.

Characteristics	Males		Females		Couple	
	*n*	%	*n*	%	*n*	%
Age, *M* (SD, range)	37.28	(6.31, 23–58)	34.78	(4.81, 22–54)		
Ethnic group						
European	213	60.9	211	60.3		
Mixed-ethnicity	97	27.7	96	27.4		
African	22	6.3	30	8.6		
Middle Eastern/Asian	11	3.1	5	1.4		
Native South American	2	0.6	0	0		
Education, high	185	52.9	239	68.3		
Job status, employed	328	93.7	290	82.9		
Income						
Low					73	20.9
Medium					186	53.1
High					91	26.0
Relationship length						
1–4 years					58	16.6
5–9 years					193	55.1
>9 years					99	28.3
Infertility type, primary					247	70.6
History of miscarriage					50	14.3
Infertility diagnosis						
Male					89	25.4
Female					86	24.6
Both male and female					59	16.9
Unexplained					116	33.1
Infertility duration						
1–2 years					122	34.9
3–4 years					113	32.3
5–6 years					74	21.1
>6 years					41	11.7
Current ART treatment, IVF					244	69.7
Previous ART failures					183	52.3

ART, assisted reproductive technology; IVF, *in vitro* fertilization.

### 3.2 Dyadic invariance of the ICQ

The proposed two-factor model for the ICQ showed an acceptable fit for both males [*χ^2^*(13) = 30.966, *p* = 0.003; RMSEA = 0.063, 90% CI 0.034–0.092, *p* = 0.205; SRMR = 0.035; CFI = 0.969] and females [*χ^2^*(13) = 36.772, *p* = 0.0004; RMSEA = 0.072, 90% CI 0.045–0.100, *p* = 0.083; SRMR = 0.051; CFI = 0.942]. The fit of the two-factor model was better than the alternative one-factor model for both males [Δ*χ^2^*(1) = 89.127, *p* < 0.001] and females [Δ*χ^2^*(1) = 84.642, *p* < 0.001]. The conceptual two-factor model was thus selected and subjected to testing of dyadic measurement invariance (Table 2).

**Table 2:** Dyadic measurement invariance of the ICQ.

Level of invariance	*df*	*χ^2^*	Δ*df*	Δ*χ^2^*	CFI	ΔCFI	RMSEA	ΔRMSEA	SRMR	ΔSRMR
Configural	64	119.944	–	^–^	0.949	–	0.050	–	0.046	–
Metric	69	126.045	5	6.101^ns^	0.948	0.001	0.049	0.001	0.049	0.003
Scalar	74	146.031	5	19.986^**^	0.935	0.013	0.053	0.004	0.051	0.002
Scalar partial^a^	73	136.802	4	10.757^*^	0.942	0.006	0.050	0.001	0.051	0.002
Strict^b^	79	140.051	6	3.249^ns^	0.945	0.003	0.047	0.003	0.053	0.002
Equal factor means	75	138.061	2	0.992^ns^	0.943	0.001	0.049	0.001	0.051	0.000

^ns^*p* > 0.05, ^*^p < 0.05, ^**^p ≤ 0.001.

^a^Intercept of item 4 freely estimated.

^b^Intercept and error variance of item 4 freely estimated.

The configural invariance model showed acceptable fit, indicating that the ICQ had a similar factor structure between couple members. The correlation between parenthood (PAR) and communication (COM) latent factors was 0.546 among males and 0.337 among females (*p* < 0.001), indicating that the two factors were moderately correlated but still independent. Correlations between males’ and females’ latent factors ranged from 0.176 (*p* < 0.05) to 0.425 (*p* < 0.001). Correlations between parallel item residuals ranged from 0.023 (*p* = 0.694) to 0.297 (*p* < 0.001). Full metric invariance was achieved, as constraining all factor loadings to be equal across couple members did not worsen model fit. This indicates that ICQ items have equivalent meaning across couple members in terms of the strength of the association between each item and its latent factor, and that the items are measuring the latent factors using the same metric scale across partners. However, full scalar invariance was not supported, as constraining all item intercepts to be equal across couple members resulted in a significant loss of fit compared to the metric invariance model, with a significant Δ*χ^2^* and a ΔCFI greater than 0.010. Subsequent analyses (Δ*χ^2^* tests with Bonferroni-corrected *α* = 0.007) revealed that the intercepts of item 4 (“The infertility problem has changed my life projects”) were non-invariant, being higher for females (0.715) than for males (0.259) [Δ*χ^2^*(1) = 9.229, *p* = 0.002]. When the equality constraint on this item’s intercept was released, the Δ*χ^2^* test remained significant (*p* = 0.029), but the changes in CFI, RMSEA, and SRMR were sufficiently small, supporting partial scalar invariance. This suggests that mean item differences across couple members are due to differences in the latent factor being measured. The strict invariance model, in which the error variances of items showing full scalar invariance were constrained to be equal across couple members, was also achieved. Therefore, the ICQ latent factors are measured with the same measurement error across both partners. Estimates for the measurement parameters from the strict invariance model are displayed in Table 3.

**Table 3:** Strict invariance model parameter estimates.

ICQ item	Factor loading	Intercept	Error variance
ICQ1. There is nothing left in life without children.	1.087 (0.051)	−0.495 (0.227)	2.748 (0.182)
ICQ2. Having a child is a central issue in my life	1.194 (0.050)	−0.808 (0.208)	1.225 (0.133)
ICQ3. Having children gives you something to live for.	0.923 (0.044)	1.303 (0.200)	1.475 (0.108)
ICQ4. The infertility problem has changed my life projects^a^.	0.796 (0.059)	0.247 (0.363)/0.703 (0.354)	4.197 (0.336)/3.273 (0.270)
ICQ5. It helps me to talk about the fact that we have trouble having kids.	1.115 (0.064)	−0.548 (0.286)	2.236 (0.233)
ICQ6. I enjoy spending time with friends who have children.	0.803 (0.058)	1.107 (0.263)	2.959 (0.200)
ICQ7. It helps me to share with other couples who have the same problem.	1.081 (0.067)	−0.558 (0.303)	2.959 (0.251)

Unstandardized estimates (standard errors) are presented; all factor loadings and error variances were significant at p < 0.001.

^a^Estimates before and after the slash refer to males and females, respectively. This item showed non-invariant intercepts in the full scalar invariance models.

Since partial scalar invariance was supported, we proceeded to test for differences in latent factor means between couple members. The addition of equality constraints on the latent factor means resulted in non-significant differences compared to a model without these constraints. Thus, males and females had similar factor means in both PAR (males: *M* = 4.713, *SE* = 0.117; females: *M* = 4.632, *SE* = 0.111) and COM (males: *M* = 4.387, *SE* = 0.087; females: *M* = 4.429, *SE* = 0.085).

### 3.3 Reliability

McDonald’s *ω* was 0.76 for males and 0.83 for females for PAR, and 0.85 for males and 0.73 for females for COM. Item-total correlations for PAR ranged from 0.38 to 0.63 among males and from 0.44 to 0.51 among females. For COM, item-total correlations ranged from 0.43 to 0.53 among males and from 0.37 to 0.48 among females.

### 3.4 Validity based on relations to other variables

For males, a significant positive correlation was observed between PAR and intrapersonal stress, with a small effect size. For females, PAR correlated significantly and positively with both intrapersonal and infertility-related stress, with small-to-medium and medium effect sizes, respectively. Among females only, there was a significant negative correlation, of small effect size, was found between COM and interpersonal infertility stress. The correlation coefficients are presented in Table 4.

**Table 4:** Correlations with IRSS (*n* = 168) and descriptive statistics (*n* = 350) of ICQ.

IRSS domain	Males		Females	
	ICQ PAR	ICQ COM	ICQ PAR	ICQ COM
IRSS Intrapersonal	0.19^*^	0.04	0.25^**^	−0.12
IRSS Interpersonal	0.08	−0.03	0.10	−0.16^*^
*M (SD)*	18.67 (6.33)	12.86 (4.90)	18.36 (5.91)	15.59 (4.95)

IRSS, Infertility Related Stress Scale; ICQ, Infertility Concern Questionnaire; PAR, parenthood; COM, communication; correlations were computed on a subsample of couples (*n* = 168) that also completed the IRSS; score range was 4–28 for ICQ PAR and 4–21 for ICQ COM. M, median; SD, standard deviation.

^*^p < 0.05, ^**^p ≤ 0.001.

As shown in Table 5, significant associations were found between females’ ICQ scores and the infertility-related characteristics of infertility type, history of miscarriage, and previous failed cycles. Specifically, female participants with secondary infertility reported significantly, slightly higher COM scores than those with primary infertility (*d* = 0.23). Females with a history of miscarriage and previous failed ART cycles reported significantly, slightly lower PAR scores than those without such histories (*d* = 0.33 and 0.32, respectively). No significant effects were observed for other infertility-related characteristics in females, and no significant associations were found among males.

**Table 5:** Associations between infertility-related characteristics and ICQ scores.

Characteristics	Males		Females	
	PAR	COM	PAR	COM
Infertility type	*F*(1,348) = 1.10	*F*(1,348) = 2.18	*F*(1,348) = 0.11	*F*(1,348) = 3.91^*^
Primary	18.88 (6.12)	13.01 (4.70)	19.31 (5.39)	12.97 (4.72)
Secondary	19.63 (5.98)	13.91 (6.30)	19.10 (5.76)	14.06 (4.66)
History of miscarriage	*F*(1,348) = 0.77	*F*(1,348) = 0.61	*F*(1,348) = 4.69^*^	*F*(1,348) = 0.01
No	18.99 (6.16)	13.36 (5.29)	19.51 (5.46)	13.30 (4.77)
Yes	19.80 (5.64)	12.74 (4.86)	17.70 (5.46)	13.22 (4.49)
Infertility diagnosis	*F*(3,346) = 0.39	*F*(3,346) = 0.55	*F*(3,346) = 1.44	*F*(3,346) = 2.50
Male	18.82 (6.63)	13.33 (4.73)	19.00 (4.93)	12.25 (4.85)
Female	19.66 (6.18)	13.84 (6.21)	19.73 (5.98)	13.16 (4.50)
Both male and female	19.24 (5.91)	12.85 (4.79)	18.07 (5.17)	14.14 (5.02)
Unexplained	18.84 (5.70)	13.03 (5.04)	19.68 (5.65)	13.75 (4.53)
Infertility duration	*F*(3,346) = 1.59	*F*(3,346) = 0.67	*F*(3,346) = 0.34	*F*(3,346) = 0.90
1–2 years	18.95 (6.19)	12.84 (5.56)	19.42 (5.29)	12.88 (4.55)
3–4 years	18.31 (6.18)	13.22 (5.18)	18.3 (5.71)	13.61 (4.77)
5–6 years	20.08 (6.17)	13.85 (5.10)	19.39 (5.71)	13.05 (4.87)
>6 years	19.98 (5.10)	13.68 (4.58)	19.63 (5.20)	14.05 (4.85)
Current ART treatment	*F*(1,348) = 3.04	*F*(1,348) = 3.28	*F*(1,348) = 1.53	*F*(1,348) = 0.10
IUI	18.25 (6.27)	12.51 (5.04)	18.70 (5.52)	13.17 (4.72)
IVF	19.48 (5.97)	13.61 (5.29)	19.49 (5.47)	13.34 (4.73)
Previous ART failures	*F*(1,348) = 0.57	*F*(1,348) = 1.17	*F*(1,348) = 8.91^**^	*F*(1,348) = 0.06
No	19.36 (5.81)	12.96 (5.81)	20.16 (5.21)	13.22 (4.73)
Yes	18.87 (4.64)	13.56 (4.64)	18.42 (5.62)	13.35 (4.73)

ICQ, Infertility Concern Questionnaire; PAR, parenthood; COM, communication; IUI, intrauterine insemination; IVF, in vitro fertilization; score range was 4–28 for PAR and 4–21 for COM.

^*^p < 0.05, ^**^p < 0.01.

## 4 Discussion

The ICQ demonstrated content and face validity, supported by a multi-stage qualitative evaluation involving expert psychologists and confirmed through focus groups with the target population of infertile individuals. Results from CFAs and measurement invariance tests supported the proposed two-correlated factor model of Parenthood (PAR) and Communication (COM) and confirmed that the ICQ functions consistently across couple members. Specifically, the ICQ demonstrated full configural and metric invariance, along with partial scalar and strict invariance. The two-factor structure was invariant across couple members, with invariant factor loadings for all items, and invariant intercepts and residual variances for 86% (6/7) of its items. Notably, this proportion aligns well with the established standards for partial measurement invariance (Steenkamp and Baumgartner, 1998). Overall, these findings indicate that the ICQ works equivalently for both male and female partners, who conceptualize PAR and COM in similar ways. For both partners, the desire for parenthood represents a central life goal that shapes their sense of purpose and future plans. Parenthood is seen not only as a source of joy and personal fulfillment but also as a key part of their identity and life projects. Infertility, in turn, has a profound impact on these aspirations, altering life trajectories and priorities. Communication, on the other hand, is viewed as an essential means of emotional support and coping, helping couples navigate the emotional and psychological challenges associated with infertility. For both partners, communication serves as a vital tool for building mutual understanding, sharing experiences, and finding relief through connections with others who face similar struggles.

Because the most stringent level of measurement invariance (i.e., strict invariance) was achieved, there is evidence that the ICQ provides equivalent precision in measurement across couple members. This allows for meaningful interpretations of between-partner comparisons on observed means and covariance structures (Millsap and Meredith, 2007). Additionally, since partial scalar invariance was reached, comparisons of ICQ latent factor means between members of infertile couples are valid. Results indicated that male and female partners had similar underlying levels of PAR and COM. This is in line with previous evidence showing that men desire parenthood as much as women (Lewis, 1988; Nitsche and Hayford, 2020; Sylvest et al., 2018; Hammarberg et al., 2017). Additionally, other studies have found no significant difference in how partners of infertile couples perceived their communication styles when dealing with stressors (Wang et al., 2024).

However, despite these broad similarities, it is important to acknowledge that men and women may experience infertility in distinct ways. The ICQ did not achieve full measurement invariance, indicating that some differences persist in how infertility-related concerns affect each partner’s life projects (item 4). This may be partially explained by the social burden disproportionately placed on women, who often face greater expectations and pressures regarding childbearing (Caddy et al., 2023; Mills, 2010).

Reliability coefficient, McDonald’s *ω*, and corrected item-total correlations, indicated acceptable levels of internal consistency for both ICQ dimensions across males and females, meeting established criteria (Gadermann et al., 2012; McNeish, 2018).

As evidence of validity based on relations to other variables, correlation analysis revealed significant, though weak, relationships between the ICQ and IRSS-BP domains. In both males and females, desire for parenthood correlated positively with the intrapersonal domain of infertility-related stress, but not with the interpersonal domain. This finding is expected, as the PAR dimension is inherently more intrapersonal in nature. The unfulfilled desire for parenthood among infertile individuals disrupts personal development, often leading to emotional turmoil, a sense of loss of control over life, and feelings of failure in identity building (Nik Hazlina et al., 2022; Alamin et al., 2020). Furthermore, the inability to have a child has been linked to reduced life satisfaction (Kiesswetter et al., 2020; Alamin et al., 2020; McQuillan et al., 2022) – one of the key components measured by the intrapersonal domain of the IRSS-BP. The absence of significant associations between PAR and interpersonal infertility stress is coherent with the notion that the stressful implications of an unachieved desire for parenthood primarily affect one’s sense of self, rather than social life (Casu and Gremigni, 2016; Casu et al., 2021; Swanson and Braverman, 2021). The significant negative correlation between COM and infertility-related stress in the interpersonal domain, observed only in women, suggests that women who are better able to communicate about their infertility challenges experience less infertility-specific stress in their relationships with family, friends, and others. For women, effective communication may help alleviate feelings of isolation, leading to stronger social support and less strain in their interpersonal interactions. In contrast, women who struggle to communicate about infertility may experience higher stress due to unresolved tensions or lack of support in these relationships. This highlights the key role communication plays in buffering the social stress women face when dealing with infertility, as also reported in previous studies (Daniluk, 1988; Chamorro et al., 2024; Kielek-Rataj et al., 2020; Wang et al., 2024). The lack of a significant association between COM and interpersonal infertility-related stress in men can be understood in light of cultural stereotypes and gender role expectations. These norms tend to place greater emphasis on motherhood and childbearing as central to women’s social identity, while men are culturally less encouraged to discuss infertility in emotional or relational terms (Taebi et al., 2021; Ying et al., 2015; Greil et al., 2010). As a result, men may not rely on communication for emotional support in the same way women do, and their communication about infertility may not significantly impact their interpersonal relationships or reduce social stress. Therefore, the COM domain, which focuses on emotional connection and support, may not serve as a strong buffer against interpersonal stress for men. These findings underscore the ICQ’s potential to guide the development of gender-sensitive interventions, tailoring psychological support strategies to the distinct communication and emotional needs of men and women.

Analyses of associations with infertility-related characteristics revealed significant associations between ICQ scores and infertility type, history of miscarriage, and previous failed cycles among women. Women with secondary infertility reported slightly higher COM scores than those with primary infertility, suggesting that they may be more inclined to use communication as a coping mechanism. Having experienced pregnancy or childbirth before, women with secondary infertility are likely more familiar with the emotional toll of reproductive challenges and may have developed stronger communication skills with their partners and social networks. This allow them to handle the emotional difficulties of infertility through open dialog, seeking emotional support and connecting with others who face similar struggles. In contrast, women with primary infertility, who have never conceived, may feel a deeper sense of stigma and societal pressure regarding motherhood, making it harder for them to seek emotional support through communication, which can lead to greater social isolation and alienation, as previously reported (Taebi et al., 2021; Raque-Bogdan and Hoffman, 2015). Female participants with previous miscarriages or failed ART cycles reported slightly lower PAR scores than those without such experiences. This finding is consistent with existing literature on psychological adaptation to infertility. As individuals repeatedly face reproductive losses, such as treatment failures or miscarriages, they may begin to revise their aspirations for parenthood, adjusting their desires in response to the growing perception that achieving their goal may be unlikely (Gray et al., 2013; Sousa-Leite et al., 2022; Boivin et al., 1995).

The ICQ has potential for both research and clinical applications. In research settings, it can serve as a valuable tool for studying the psychological and relational aspects of infertility, particularly in exploring how parenthood aspirations and communication around infertility interact. It can also contribute to evaluating interventions aimed at improving the personal and social adjustment of infertile couples by tracking changes in these dimensions over time. Moreover, researchers can use the ICQ to compare different populations and identify key factors influencing emotional resilience in infertility contexts.

In clinical practice, the ICQ can be integrated into routine assessments to provide a comprehensive understanding of infertile couples’ emotional and communicative concerns. Its practical integration into routine assessments could occur during the initial stages of a couple’s engagement with assisted reproduction services, allowing clinicians to quickly gather valuable insights and tailor support accordingly. This allows healthcare providers to tailor interventions more effectively, addressing specific needs and improving patient outcomes. The use of the ICQ does not require specialized training; any healthcare provider familiar with diagnostic and psychometric scales would be sufficiently equipped to administer it. Furthermore, its scoring system is intuitive and facilitates the interpretation of the results obtained.

By identifying distinct dimensions of parenthood desire and infertility-related communication skills, the ICQ can help counselors and therapists develop personalized counseling strategies, enhancing the emotional support offered to couples as they navigate the psychological challenges of infertility. The ICQ also highlights the critical role of communication in managing infertility. Healthcare providers can use insights from the questionnaire to design targeted communication training programs that foster better mutual support and understanding between partners. Furthermore, it can be used in support groups, identifying common concerns and communication challenges among participants. Group leaders can then structure discussions and activities that address these issues in meaningful ways.

Finally, the ICQ can inform the development of policies and programs that address the psychosocial aspects of infertility. This can lead to more holistic reproductive health services that ensure emotional and communicative needs are adequately met.

In summary, the ICQ can be a versatile tool in research, clinical practice, group counseling, and policy development, helping healthcare providers support couples by addressing both their desire for parenthood and the vital role of communication in their infertility journey.

### 4.1 Limitations and future directions

While this study provides valuable preliminary insights into the psychometric functioning and usability of the ICQ, several limitations should be acknowledged. First, Brazil is characterized by substantial cultural and socioeconomic diversity across its regions, which may limit the generalizability of the findings. Although the use of a single measure of infertility-related stress provided useful evidence for construct validity, it may not fully capture the multifaceted nature of infertility-related experiences. Future research should incorporate a broader range of validated instruments—such as those assessing mental health, self-efficacy, and perceived social support from both partners and the wider social network—to strengthen the robustness and comprehensiveness of the validation process. In particular, construct validity for the ICQ could be further examined in future studies through the use of tools such as the Decision Regret Scale (Xu et al., 2020), given the significant impact that assisted reproductive treatment decisions can have on patients’ lives.

Additionally, more evidence is needed in relation to variables that are especially relevant to the experience of male partners, such as perceived social stigma, self-image, and beliefs about masculinity (Hanna and Gough, 2020; Hughes et al., 2024). Test–retest reliability was not assessed in this study, representing a limitation in evaluating the instrument’s stability over time. Longitudinal studies could address this gap by administering the ICQ at multiple stages of the ART process, thus assessing both its temporal stability and its sensitivity to clinically meaningful changes (e.g., treatment failures, pregnancy, or treatment pauses). Future research should also explore how the ICQ relates to other psychological measures and sociodemographic or clinical characteristics, potentially enhancing its psychometric robustness and clinical applicability. Moreover, future investigations could examine the specific role of gender in communication as a coping strategy for infertility—potentially through the integration of qualitative interviews or evaluations of couple communication quality.

Finally, the sample was recruited from a single reproductive health center, which may restrict the external validity of the results. Expanding future studies to include participants from multiple clinics and geographically diverse regions would enhance the generalizability and applicability of the findings to broader populations.

## 5 Conclusion

The findings of this study provide initial evidence of the validity and reliability of the ICQ as a tool for rapidly assessing the desire for parenthood and communication about infertility, both within and outside the couple. Notably, the dimensionality and measurement properties of the ICQ were invariant across male and female partners of infertile couples. With support for (partial) scalar and strict invariance, Brazilian researchers and clinicians can confidently use the ICQ to explore potential differences in desire for parenthood and infertility-related communication issues between male and female members of infertile couples, assured that any observed differences reflect true, conceptually meaningful variations rather than measurement biases. Future research should further investigate whether dyadic invariance holds across different types of infertility (i.e., primary vs. secondary) and ART treatments (e.g., intrauterine insemination or *in vitro* fertilization), to enhance our understanding of how infertility concerns manifest in different clinical contexts. Additionally, more studies are required to gather further evidence of the ICQ’s validity based on relations to relevant variables and to assess its temporal stability and sensitivity to change.

## Data Availability

The datasets presented in this study can be found in online repositories. The names of the repository/repositories and accession number(s) can be found: https://doi.org/10.17605/OSF.IO/2QYPJ.

## References

[ref1] Abbey A. Halman L. J. Andrews F. M. (1992). Psychosocial, treatment, and demographic predictors of the stress associated with infertility. Fertil. Steril. 57, 122–128. doi: 10.1016/S0015-0282(16)54787-6, PMID: 1730305

[ref2] Alamin S. Allahyari T. Ghorbani B. Sadeghitabar A. Karami M. T. (2020). Failure in identity building as the main challenge of infertility: a qualitative study. J. Reprod. Infertil. 21, 49–58. 32175265 PMC7048687

[ref3] Assaysh-Oberg S. Borneskog C. Ternstrom E. (2023). Women’s experience of infertility and treatment – a silent grief and failed care and support. Sex. Reprod. Healthc. 37:100879. doi: 10.1016/j.srhc.2023.100879, PMID: 37356208

[ref4] Baiocco R. Laghi F. (2013). Sexual orientation and the desires and intentions to become parents. J. Fam. Stud. 19:9. doi: 10.5172/jfs.2013.19.1.90

[ref5] Batz F. Lermer E. Lech S. O’Malley G. Zati Zehni A. Zenz-Spitzweg D.. (2023). Publisher correction: the psychological burden of COVID-19 on the desire for parenthood in minoritized sexual identities: a study on depressive symptoms and family planning in Germany. BMC Public Health 23:609. doi: 10.1186/s12889-023-15385-5 36997987 PMC10062679

[ref6] Beaujouan E. (2020). Latest-late fertility? Decline and resurgence of late parenthood across the low-fertility countries. Popul. Dev. Rev. 46, 219–247. doi: 10.1111/padr.12334, PMID: 32733116 PMC7384131

[ref7] Beck A. T. Ward C. H. Mendelson M. Mock J. Erbaugh J. (1961). An inventory for measuring depression. Arch. Gen. Psychiatry 4, 561–571. doi: 10.1001/archpsyc.1961.01710120031004, PMID: 13688369

[ref8] Bernstein J. Potts N. Mattox J. H. (1985). Assessment of psychological dysfunction associated with infertility. J. Obstetric Gynecol. Neonatal. Nurs. 14, S63–S66. doi: 10.1111/j.1552-6909.1985.tb02803.x, PMID: 3852872

[ref9] Biggs S. N. Halliday J. Hammarberg K. (2024). Psychological consequences of a diagnosis of infertility in men: a systematic analysis. Asian J. Androl. 26, 10–19. doi: 10.4103/aja202334, PMID: 37695221 PMC10846829

[ref10] Boivin J. Bunting L. Collins J. A. Nygren K. G. (2007). International estimates of infertility prevalence and treatment-seeking: potential need and demand for infertility medical care. Hum. Reprod. 22, 1506–1512. doi: 10.1093/humrep/dem046, PMID: 17376819

[ref11] Boivin J. Oguz M. Duong M. Cooper O. Filipenko D. Markert M.. (2023). Emotional reactions to infertility diagnosis: thematic and natural language processing analyses of the 1000 dreams survey. Reprod. Biomed. Online 46, 399–409. doi: 10.1016/j.rbmo.2022.08.107, PMID: 36463078

[ref12] Boivin J. Takefman J. Braverman A. (2011). The fertility quality of life (Ferti QoL) tool: development and general psychometric properties. Hum. Reprod. 26, 2084–2091. doi: 10.1093/humrep/der171, PMID: 21665875 PMC3137391

[ref13] Boivin J. Takefman J. E. Tulandi T. Brender W. (1995). Reactions to infertility based on extent of treatment failure. Fertil. Steril. 63, 801–807. doi: 10.1016/S0015-0282(16)57485-8, PMID: 7890066

[ref14] Bose S. Roy B. Umesh S. (2021). Marital duration, and fertility-related stress as predictors of quality of life: gender differences among primary infertile couples. J. Hum. Reproduct. Sci. 14, 184–190. doi: 10.4103/jhrs.jhrs_233_20, PMID: 34316235 PMC8279062

[ref15] Braverman A. M. Davoudian T. Levin I. K. Bocage A. Wodoslawsky S. (2024). Depression, anxiety, quality of life, and infertility: a global lens on the last decade of research. Fertil. Steril. 121, 379–383. doi: 10.1016/j.fertnstert.2024.01.013, PMID: 38224730

[ref16] Caddy C. Temple-Smith M. Coombe J. (2023). Who does what? Reproductive responsibilities between heterosexual partners. Cult. Health Sex. 25, 1640–1658. doi: 10.1080/13691058.2023.2173800, PMID: 36752653

[ref17] Canada A. L. Schover L. R. (2012). The psychosocial impact of interrupted childbearing in long-term female cancer survivors. Psycho-Oncology 21, 134–143. doi: 10.1002/pon.1875, PMID: 22271533 PMC3123665

[ref18] Casu G. Gremigni P. (2016). Screening for infertility-related stress at the time of initial infertility consultation: psychometric properties of a brief measure. J. Adv. Nurs. 72, 693–706. doi: 10.1111/jan.12830, PMID: 26466927

[ref19] Casu G. Zaia V. Montagna E. de Padua Serafim A. Bianco B. Barbosa C. P.. (2021). The infertility-related stress scale: validation of a Brazilian-Portuguese version and measurement invariance across Brazil and Italy. Front. Psychol. 12:784222. doi: 10.3389/fpsyg.2021.784222, PMID: 35095671 PMC8792459

[ref20] Cetre S. Clark A. E. Senik C. (2016). Happy people have children: choice and self-selection into parenthood. Eur. J. Popul. 32, 445–473. doi: 10.1007/s10680-016-9389-x, PMID: 28713186 PMC5505668

[ref21] Chamorro P. P. Herruzo J. Pino M. J. Casas-Rosal J. C. (2024). Coping, social support and medical factors on psychosocial impact in couples experiencing infertility. J. Sex Marital Ther. 50, 197–215. doi: 10.1080/0092623X.2023.2269983, PMID: 37867461

[ref22] Chu X. Geng Y. Zhang R. Guo W. (2021). Perceived social support and life satisfaction in infertile women undergoing treatment: a moderated mediation model. Front. Psychol. 12:651612. doi: 10.3389/fpsyg.2021.651612, PMID: 34122236 PMC8194393

[ref1000] Chen F. F. (2007). Sensitivity of Goodness of Fit Indexes to Lack of Measurement Invariance. Structural Equation Modeling: A Multidisciplinary Journal. 14, 464–504. doi: 10.1080/10705510701301834

[ref23] Claxton S. E. DeLuca H. K. van Dulmen M. H. M. (2015). Testing psychometric properties in dyadic data using confirmatory factor analysis: current practices and recommendations. Test. Psychom. Methodol. Appl. Psychol. 22:18. doi: 10.4473/TPM22.2.2

[ref24] Cohen J. (1988). Statistical power analysis for the behavioral sciences. 2nd Edn. New York: Lawrence Erlbaum Associates.

[ref25] Daniluk J. C. (1988). Infertility: intrapersonal and interpersonal impact. Fertil. Steril. 49, 982–990. doi: 10.1016/S0015-0282(16)59948-8, PMID: 3371493

[ref26] Gadermann A. M. Guhn M. Zumbo B. D. (2012). Estimating or estimating ordinal r dinal reliability for like liability for liker ert-type and or t-type and ordinal item dinal itemrresponse data: a conceptual, empirical, and response data: a conceptual, empirical, and practical guide. Pract. Assess. Res. Evaluatio. 17:13. doi: 10.7275/n560-j767

[ref27] Gameiro S. Boivin J. Dancet E. de Klerk C. Emery M. Lewis-Jones C.. (2015). ESHRE guideline: routine psychosocial care in infertility and medically assisted reproduction-a guide for fertility staff. Hum. Reprod. 30, 2476–2485. doi: 10.1093/humrep/dev177, PMID: 26345684

[ref28] Gameiro S. Finnigan A. (2017). Long-term adjustment to unmet parenthood goals following ART: a systematic review and meta-analysis. Hum. Reprod. Update 23, 322–337. doi: 10.1093/humupd/dmx001, PMID: 28164236

[ref29] Gray E. Evans A. Reimondos A. (2013). Childbearing desires of childless men and women: when are goals adjusted? Adv. Life Course Res. 18, 141–149. doi: 10.1016/j.alcr.2012.09.003, PMID: 24796265

[ref30] Greil A. L. Slauson-Blevins K. McQuillan J. (2010). The experience of infertility: a review of recent literature. Sociol. Health Illn. 32, 140–162. doi: 10.1111/j.1467-9566.2009.01213.x, PMID: 20003036 PMC3383794

[ref31] Hammarberg K. Collins V. Holden C. Young K. McLachlan R. (2017). Men’s knowledge, attitudes and behaviours relating to fertility. Hum. Reprod. Update 23, 458–480. doi: 10.1093/humupd/dmx005, PMID: 28333354

[ref32] Hanna E. Gough B. (2020). The social construction of male infertility: a qualitative questionnaire study of men with a male factor infertility diagnosis. Sociol. Health Illn. 42, 465–480. doi: 10.1111/1467-9566.13038, PMID: 31773768

[ref33] Harkness S. Super C. M. (2002). Culture and parenting. Handbook of parenting: Biology and ecology of parenting, vol. 2. 2nd Edn. Mahwah, NJ, US: Lawrence Erlbaum Associates Publishers, 253–280.

[ref34] Ho T. T. T. Le M. T. Truong Q. V. Nguyen V. Q. H. Cao N. T. (2020). Psychological burden in couples with infertility and its association with sexual dysfunction. Sex. Disabil. 38:10. doi: 10.1007/s11195-019-09612-4

[ref35] Hu L. Bentler P. M. (1999). Cutoff criteria for fit indexes in covariance structure analysis: conventional criteria versus new alternatives. Struct. Equ. Model. 6, 1–55. doi: 10.1080/10705519909540118

[ref36] Hughes S. Oswald F. Pedersen C. L. (2024). The virility-fertility tradeoff: effects of fatherhood on (precarious) masculinity, sexual esteem, and sexual depression. Psychol. Sex. 16:6863. doi: 10.1080/19419899.2024.2366863

[ref37] Jain M. Singh M. (2024). Assisted reproductive technology (ART) techniques. Treasure Island (FL): Stat Pearls. 35015434

[ref38] Jansen C. Kuhlmann E. Scharli P. Schick M. Ditzen B. Langer L.. (2022). ‘A sorrow shared …’: a qualitative content analysis of what couples with recurrent miscarriages expect from one another and their families and friends. Hum Reprod Open. 2022:hoac032. doi: 10.1093/hropen/hoac032, PMID: 35928048 PMC9345061

[ref39] Järvinen T. (1998). The family, culture and educational choices. Young 6, 2–14. doi: 10.1177/110330889800600201, PMID: 40441278

[ref40] Kielek-Rataj E. Wendolowska A. Kalus A. Czyzowska D. (2020). Openness and communication effects on relationship satisfaction in women experiencing infertility or miscarriage: a dyadic approach. Int. J. Environ. Res. Public Health 17:5721. doi: 10.3390/ijerph17165721, PMID: 32784727 PMC7459658

[ref41] Kiesswetter M. Marsoner H. Luehwink A. Fistarol M. Mahlknecht A. Duschek S. (2020). Impairments in life satisfaction in infertility: associations with perceived stress, affectivity, partnership quality, social support and the desire to have a child. Behav. Med. 46, 130–141. doi: 10.1080/08964289.2018.1564897, PMID: 30726170

[ref42] Kitchen H. Aldhouse N. Trigg A. Palencia R. Mitchell S. (2017). A review of patient-reported outcome measures to assess female infertility-related quality of life. Health Qual. Life Outcomes 15:86. doi: 10.1186/s12955-017-0666-0, PMID: 28449717 PMC5408488

[ref43] Kline R. (2016). Principles and practice of structural equation modeling. 4th Edn. New York, NY: The Guilford Press.

[ref44] Lewis J. M. (1988). The transition to parenthood: II. Stability and change in marital structure. Fam. Process 27, 273–283. doi: 10.1111/j.1545-5300.1988.00273.x, PMID: 3224698

[ref45] Little T. D. Slegers D. W. Card N. A. (2006). A non-arbitrary method of identifying and scaling latent variables in SEM and MACS models. Struct. Equ. Modeling 13, 59–72. doi: 10.1207/s15328007sem1301_3

[ref46] Luca G. Parrettini S. Sansone A. Calafiore R. Jannini E. A. (2021). The Inferto-sex syndrome (ISS): sexual dysfunction in fertility care setting and assisted reproduction. J. Endocrinol. Investig. 44, 2071–2102. doi: 10.1007/s40618-021-01581-w, PMID: 33956331 PMC8421318

[ref47] Massarotti C. Gentile G. Ferreccio C. Scaruffi P. Remorgida V. Anserini P. (2019). Impact of infertility and infertility treatments on quality of life and levels of anxiety and depression in women undergoing in vitro fertilization. Gynecol. Endocrinol. 35, 485–489. doi: 10.1080/09513590.2018.1540575, PMID: 30612477

[ref48] McNeish D. (2018). Thanks coefficient alpha, we’ll take it from here. Psychol. Methods 23, 412–433. doi: 10.1037/met0000144, PMID: 28557467

[ref49] McQuillan J. Passet-Wittig J. Greil A. L. Bujard M. (2022). Is perceived inability to procreate associated with life satisfaction? Evidence from a German panel study. Reprod Biomed Soc Online. 14, 87–100. doi: 10.1016/j.rbms.2021.09.004, PMID: 34877417 PMC8627902

[ref50] Meijering J. V. Kampen J. K. Tobi H. (2013). Quantifying the development of agreement among experts in Delphi studies. Technol. Forecast. Soc. Change 80, 1607–1614. doi: 10.1016/j.techfore.2013.01.003

[ref51] Miller W. B. (1995). Childbearing motivation and its measurement. J. Biosoc. Sci. 27, 473–487. doi: 10.1017/S0021932000023087, PMID: 7593054

[ref52] Mills M. (2010). Gender roles, gender (in)equality and fertility: an empirical test of five gender equity indices. Can. Stud. Popul. 37:30. doi: 10.25336/P6131Q

[ref53] Millsap R. E. Meredith W. (2007). “Factorial invariance: historical perspectives and new problems” in Factor analysis at 100: Historical developments and future directions. eds. Cudeck R. Mac Callum R. C.. 1st ed (London: Lawrence Erlbaum Associates Publishers), 131–152.

[ref54] Miscioscia M. Blavier A. Pagone P. R. Simonelli A. (2017). The desire of parenthood: intuitive co-parental behaviors and quality of couple relationship among Italian and Belgian same-sex and opposite-sex couples. Front. Psychol. 8:110. doi: 10.3389/fpsyg.2017.00110, PMID: 28261120 PMC5306294

[ref55] Newton C. R. Sherrard W. Glavac I. (1999). The fertility problem inventory: measuring perceived infertility-related stress. Fertil. Steril. 72, 54–62. doi: 10.1016/S0015-0282(99)00164-8, PMID: 10428148

[ref56] Nik Hazlina N. H. Norhayati M. N. Shaiful Bahari I. Nik Muhammad Arif N. A. (2022). Worldwide prevalence, risk factors and psychological impact of infertility among women: a systematic review and meta-analysis. BMJ Open 12:e057132. doi: 10.1136/bmjopen-2021-057132, PMID: 35354629 PMC8968640

[ref57] Nitsche N. Hayford S. R. (2020). Preferences, partners, and parenthood: linking early fertility desires, marriage timing, and achieved fertility. Demography 57, 1975–2001. doi: 10.1007/s13524-020-00927-y, PMID: 33179200 PMC7732806

[ref58] Nunnally J. C. Bernstein I. H. (1994). Psychometric theory. 3rd Edn. New York: McGraw-Hill, Inc.

[ref59] O’Connell S. B. L. Gelgoot E. N. Grunberg P. H. Schinazi J. Da Costa D. Dennis C. L.. (2021). ‘I felt less alone knowing i could contribute to the forum’: psychological distress and use of an online infertility peer support forum. Health Psychol. Behav. Med. 9, 128–148. doi: 10.1080/21642850.2021.1884556, PMID: 34104553 PMC8158233

[ref60] Omani-Samani R. Maroufizadeh S. Almasi-Hashiani A. Sepidarkish M. Amini P. (2019). The WHO-5 well-being index: a validation study in people with infertility. Iran. J. Public Health 48, 2058–2064. 31970105 PMC6961185

[ref61] Ozturk R. Herbell K. Morton J. Bloom T. (2021). “the worst time of my life”: treatment-related stress and unmet needs of women living with infertility. J. Community Psychol. 49, 1121–1133. doi: 10.1002/jcop.22527, PMID: 33616236 PMC8324009

[ref62] Pedro J. Sobral M. P. Mesquita-Guimaraes J. Leal C. Costa M. E. Martins M. V. (2017). Couples’ discontinuation of fertility treatments: a longitudinal study on demographic, biomedical, and psychosocial risk factors. J. Assist. Reprod. Genet. 34, 217–224. doi: 10.1007/s10815-016-0844-8, PMID: 27900611 PMC5306409

[ref63] Präg P. Mills M. Tanturri M. Monden C. Pison G. (2017). The demographic consequences of assisted reproductive technologies. Oxford: Hal Open Science.

[ref64] Raque-Bogdan T. L. Hoffman M. A. (2015). The relationship among infertility, self-compassion, and well-being for women with primary or secondary infertility. Psychol. Women Q. 39, 484–496. doi: 10.1177/0361684315576208

[ref65] Ribeiro A. C. (2007) Adaptação do inventário de problemas de fertilidade para homens e mulheres inférteis [Doctoral]: Universidade de São Paulo;

[ref66] Ryu E. (2011). Effects of skewness and kurtosis on normal-theory based maximum likelihood test statistic in multilevel structural equation modeling. Behav. Res. Methods 43, 1066–1074. doi: 10.3758/s13428-011-0115-7, PMID: 21671139

[ref67] Schmidt L. (2006). Infertility and assisted reproduction in Denmark. Epidemiology and psychosocial consequences. Dan. Med. Bull. 53, 390–417. 17150146

[ref68] Schmidt L. Holstein B. E. Christensen U. Boivin J. (2005). Communication and coping as predictors of fertility problem stress: cohort study of 816 participants who did not achieve a delivery after 12 months of fertility treatment. Hum. Reprod. 20, 3248–3256. doi: 10.1093/humrep/dei193, PMID: 16006458

[ref69] Sharma A. Shrivastava D. (2022). Psychological problems related to infertility. Cureus 14:e30320. doi: 10.7759/cureus.30320, PMID: 36407201 PMC9661871

[ref70] Shen Q. Wang B. He T. Li S. Peng E. Lei J. (2024). Factors associated with discontinuation in fertility treatment: a systematic scoping review. J. Assist. Reprod. Genet. 41, 409–421. doi: 10.1007/s10815-023-02982-x, PMID: 37987953 PMC10894784

[ref71] Shreffler K. M. Gallus K. L. Peterson B. Greil A. L. (2020). “Couples and infertility” in The Handbook of Systemic Family Therapy. eds. Wampler K. Miller R. Seedall R. McWey L. Blow A. Rastogi M. (Hoboken: John Wiley and Sons) 385–406.

[ref72] Sormunen T. Aanesen A. Fossum B. Karlgren K. Westerbotn M. (2018). Infertility-related communication and coping strategies among women affected by primary or secondary infertility. J. Clin. Nurs. 27, e335–e344. doi: 10.1111/jocn.13953, PMID: 28677273

[ref73] Sousa-Leite M. Fernandes M. Reis S. Costa R. Figueiredo B. Gameiro S. (2022). Feasibility and acceptability of psychosocial care for unsuccessful fertility treatment. Health Expect. 25, 2902–2913. doi: 10.1111/hex.13598, PMID: 36128606 PMC9700180

[ref74] Starc A. Trampus M. Pavan Jukic D. Rotim C. Jukic T. Polona M. A. (2019). Infertility and sexual dysfunctions: a systematic literature review. Acta Clin. Croat. 58, 508–515. doi: 10.20471/acc.2019.58.03.15, PMID: 31969764 PMC6971809

[ref75] Steenkamp J. B. E. M. Baumgartner H. (1998). Assessing measurement invariance in cross-national consumer research. J. Consum. Res. 25, 78–90.

[ref76] Swanson A. Braverman A. M. (2021). Psychological components of infertility. Fam. Court. Rev. 59, 67–82. doi: 10.1111/fcre.12552

[ref77] Sylvest R. Koert E. Birch Petersen K. Malling G. M. H. Hald F. Nyboe Andersen A.. (2018). Attitudes towards family formation among men attending fertility counselling. Reprod Biomed Soc Online. 6, 1–9. doi: 10.1016/j.rbms.2018.06.001, PMID: 30182067 PMC6120434

[ref78] Taebi M. Kariman N. Montazeri A. Alavi Majd H. (2021). Infertility stigma: a qualitative study on feelings and experiences of infertile women. Int. J. Fertil. Steril. 15, 189–196. doi: 10.22074/IJFS.2021.139093.1039, PMID: 34155865 PMC8233927

[ref79] Tang N. Jia Y. Zhao Q. Liu H. Li J. Zhang H.. (2022). Influencing factors of dyadic coping among infertile women: a path analysis. Front. Psych. 13:830039. doi: 10.3389/fpsyt.2022.830039, PMID: 35418892 PMC8995970

[ref80] Tavousi S. A. Behjati M. Milajerdi A. Mohammadi A. H. (2022). Psychological assessment in infertility: a systematic review and meta-analysis. Front. Psychol. 13:961722. doi: 10.3389/fpsyg.2022.961722, PMID: 36389481 PMC9650266

[ref81] van Balen F. Trimbos-Kemper T. C. (1995). Involuntarily childless couples: their desire to have children and their motives. J. Psychosom. Obstet. Gynaecol. 16, 137–144. doi: 10.3109/01674829509024462, PMID: 8528380

[ref82] Vignoli D. Minello A. Bazzani G. Matera C. Rapallini C. (2022). Narratives of the future affect fertility: evidence from a laboratory experiment. Eur. J. Popul. 38, 93–124. doi: 10.1007/s10680-021-09602-3, PMID: 35370526 PMC8924345

[ref83] Wang Q. Jia D. Gao Y. Zhou M. Zhao X. Qin R.. (2024). Relationship between stigma and infertility-related stress among couples undergoing AID: the mediating role of communication patterns. Stress. Health 40:e3412. doi: 10.1002/smi.3412, PMID: 38651677

[ref84] WHO (1995). The World Health Organization quality of life assessment (WHOQOL): position paper from the World Health Organization. Soc. Sci. Med. 41, 1403–1409. 8560308 10.1016/0277-9536(95)00112-k

[ref85] WHO, W. H. O. (2006). Reproductive health indicators: guidelines for their generation, interpretation and analysis for global monitoring (WHO, Ed.). Geneve: WHO Press.

[ref86] Xie Y. Ren Y. Niu C. Zheng Y. Yu P. Li L. (2022). The impact of stigma on mental health and quality of life of infertile women: a systematic review. Front. Psychol. 13:1093459. doi: 10.3389/fpsyg.2022.1093459, PMID: 36698573 PMC9869765

[ref87] Xu R. H. Zhou L. M. Wong E. L. Wang D. Chang J. H. (2020). Psychometric evaluation of the Chinese version of the decision regret scale. Front. Psychol. 11:583574. doi: 10.3389/fpsyg.2020.583574, PMID: 33424697 PMC7793926

[ref88] Ying L. Y. Wu L. H. Loke A. Y. (2015). Gender differences in experiences with and adjustments to infertility: a literature review. Int. J. Nurs. Stud. 52, 1640–1652. doi: 10.1016/j.ijnurstu.2015.05.004, PMID: 26021885

[ref89] Zurlo M. C. Cattaneo Della Volta M. F. Vallone F. (2023). Paths towards parenthood after repeated treatment failures: a comparative study on predictors of psychological health outcomes in infertile couples persisting in treatments or opting for adoption. Front. Psychol. 14:1147926. doi: 10.3389/fpsyg.2023.1147926, PMID: 37342643 PMC10277654

